# Profiling the Cross Reactivity of Ubiquitin with the Nedd8 Activating Enzyme by Phage Display

**DOI:** 10.1371/journal.pone.0070312

**Published:** 2013-08-01

**Authors:** Bo Zhao, Keya Zhang, Karan Bhuripanyo, Chan Hee J. Choi, Eric B. Villhauer, Heng Li, Ning Zheng, Hiroaki Kiyokawa, Hermann Schindelin, Jun Yin

**Affiliations:** 1 Department of Chemistry, University of Chicago, Chicago, Illinois, United States of America; 2 Department of Pharmacology, Howard Hughes Medical Institute, University of Washington, Seattle, Washington, United States of America; 3 Department of Pharmacology and Biological Chemistry, Northwestern University Feinberg School of Medicine, Chicago, Illinois, United States of America; 4 Rudolf Virchow Center for Experimental Biomedicine and Institute for Structural Biology, University of Würzburg, Würzburg, Germany; George Washington University, United States of America

## Abstract

The C-terminal peptides of ubiquitin (UB) and UB-like proteins (UBLs) play a key role in their recognition by the specific activating enzymes (E1s) to launch their transfer through the respective enzymatic cascades thus modifying cellular proteins. UB and Nedd8, a UBL regulating the activity of cullin-RING UB ligases, only differ by one residue at their C-termini; yet each has its specific E1 for the activation reaction. It has been reported recently that UAE can cross react with Nedd8 to enable its passage through the UB transfer cascade for protein neddylation. To elucidate differences in UB recognition by UAE and NAE, we carried out phage selection of a UB library with randomized C-terminal sequences based on the catalytic formation of UB∼NAE thioester conjugates. Our results confirmed the previous finding that residue 72 of UB plays a “gate-keeping” role in E1 selectivity. We also found that diverse sequences flanking residue 72 at the UB C-terminus can be accommodated by NAE for activation. Furthermore heptameric peptides derived from the C-terminal sequences of UB variants selected for NAE activation can function as mimics of Nedd8 to form thioester conjugates with NAE and the downstream E2 enzyme Ubc12 in the Nedd8 transfer cascade. Once the peptides are charged onto the cascade enzymes, the full-length Nedd8 protein is effectively blocked from passing through the cascade for the critical modification of cullin. We have thus identified a new class of inhibitors of protein neddylation based on the profiles of the UB C-terminal sequences recognized by NAE.

## Introduction

**Figure 1:**
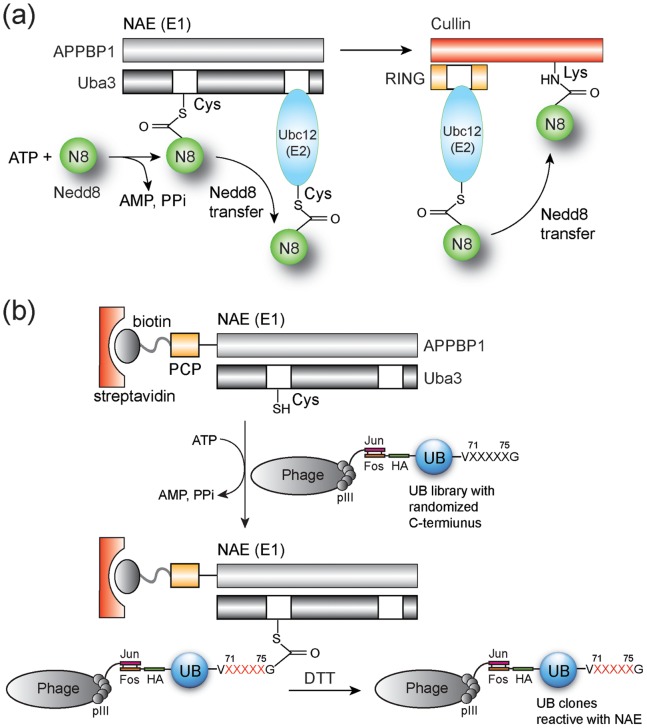
The Nedd8 transfer cascade and phage selection of UB variants for NAE activation. (a) Nedd8 is first activated by heterodimeric NAE composed of the APPBP1 and Uba3 subunits to form a Nedd8∼NAE thioester conjugate followed by the transfer of Nedd8 to the E2 enzyme Ubc12. The Nedd8∼Ubc12 conjugate is then bound to the cullin-RING complex for cullin modification by Nedd8. (b) For phage selection of UB variants reactive with NAE, a PCP-NAE fusion was labeled with biotin and immobilized on a streptavidin plate. Phage library displaying UB with randomized C-terminal sequences was added to the streptavidin plate with ATP to allow UB variants to form thioester conjugates with NAE. Phage displaying NAE reactive UB variants were eluted by cleaving the thioester bond in the UB∼NAE conjugate by DTT.

Nedd8 is a ubiquitin-like protein (UBL) that covalently modifies the cullin subunits of the cullin-RING complexes to turn on their activities as E3 ubiquitin (UB) ligases (Figure 1a) [1], [2], [3], [4], [5], [6]. The E1 enzyme specific for Nedd8, also known as Nedd8 activating enzyme (NAE), catalyzes the condensation of ATP with the C-terminal carboxylate of Nedd8 to form a Nedd8-AMP conjugate [5], [7], [8]. The activated Nedd8 is then captured by a catalytic Cys residue of NAE to form a Nedd8∼NAE thioester conjugate (“∼” designates the thioester linkage). Subsequently a thioester exchange reaction leads to the transfer of Nedd8 from NAE to the E2 enzyme that carries Nedd8 to cullin for its modification [9]. UB has its own set of one or two E1s and several dozen E2s that activate and transfer UB following the same mechanism; the E1 enzymes specific for UB (UB activation enzyme or UAE) catalyze the formation of UB∼E1 conjugates followed by UB transfer to E2s to form UB∼E2 conjugates. The UB∼E2 conjugates are then bound to the E3 enzymes such as the cullin-RING complexes to deliver UB to the substrate proteins recruited by the E3s [10], [11].

Previously it was thought that the E1–E2 cascade for Nedd8 modification and the E1–E2–E3 cascades for UB modification do not cross react. However, it was recently reported that Nedd8 can enter the E1–E2–E3 cascades for UB transfer that results in the neddylation of the proteins targeted by E3 UB ligases [12], [13], [14]. It was also found that mutations at the UB C-terminus enable UB to be efficiently activated by NAE for its loading on Ubc12, the E2 that is the exclusive carrier of Nedd8 [15], [16]. These observations suggest that the enzymatic cascades for Nedd8 and UB transfer maybe intertwined for the crossover of protein ubiquitination and neddylation pathways in the cell.

Nedd8 and UB both contain 76 residues and share the highest sequence homology among all the UBLs with 57% of the residues being identical and 76% of the residues being similar to each other [17], [18]. As expected the two proteins also adopt very similar structures featuring the β-grasp fold [19]. Their E1 enzymes are also homologous to each other in both peptide sequences and crystal structures, except that NAE is a heterodimer of two subunits, APPBP1 and Uba3, while UAE is composed of a single chain [8], [20], [21]. The crystal structures of the E1s bound to their cognate Nedd8 and UB proteins show that Nedd8 and UB are docked to the E1s in a similar mode with their C-terminal peptide extending into the adenylation domains of the E1s to reach to the ATP molecule bound to the E1s [20], [22]. The C-terminal peptides of Nedd8 and UB have the sequences ^71^LALRGG^76^ and ^71^LRLRGG^76^, respectively, with just one residue at position 72 being different. Both Ala72 of Nedd8 and Arg72 of UB have matching residues in NAE and UAE for complementary interactions. It was found that when Ala72 of Nedd8 was mutated to Arg, the Nedd8 mutant acquired a 65-fold increase in affinity with UAE and can be activated by UAE [17]. Vice versa, when Arg72 in UB was mutated to Ala or Leu, UB can be efficiently activated by NAE [15], [16]. Thus, residues at position 72 of Nedd8 and UB provide a “gating mechanism” to ensure specific pairing of Nedd8 and UB with their cognate E1s in the activation reactions [23].

In this study, we further elucidated differences in NAE and UAE recognizing the C-terminal sequence of UB. We carried out phage selection of a UB library to generate a profile of UB C-terminal sequences that are preferentially recognized by NAE for UB activation. We found that hydrophobic residues such as Ala, Val, Leu, and polar residues such as Ser and Gln can replace Arg72 of UB to enable its activation by NAE. NAE can also accommodate diverse nonnative sequences flanking residue 72 for UB activation. Based on the sequence profile of the UB C-terminus for NAE activation, we identified short peptides that can be activated by NAE and form thioester conjugates with NAE and Ubc12. We further showed that the loading of these peptides on NAE and Ubc12 can effectively block Nedd8 transfer through the NAE-Ubc12 cascade for cullin modification.

## Results

### Phage Selection of UB Variants that can be Activated by NAE

We profiled the C-terminal sequences of UB that are catalytically active with NAE based on a phage display method we developed to engineer UB recognition by UAE (Figure 1b) [24]. In this method we displayed a UB library with randomized C-terminal sequences on the surface of the M13 phage. The phage library was added to a streptavidin plate immobilized with biotin-labeled NAE. Phage particles displaying UB variants recognized by NAE were covalently bound to the plate due to the formation of a UB∼NAE conjugate. Upon elution by dithiothreitol (DTT) to break the thioester linkage, the selected phage clones were amplified for the next round of selection. After multiple selections, the enriched phage clones were sequenced to identify the C-terminal sequences of UB variants that are reactive with NAE (Figure 1b).

**Figure 2:**
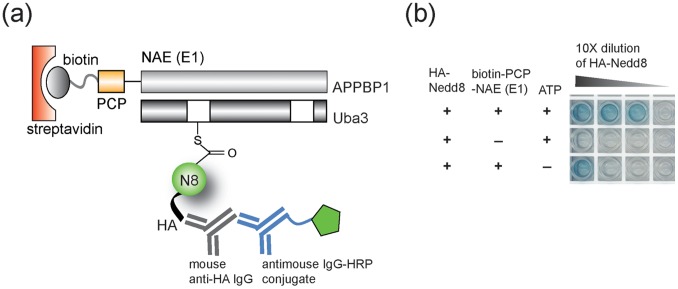
Measuring the activity of biotin-labeled PCP-NAE fusion protein immobilized on the streptavidin plate by ELISA. (a) Assaying the formation of Nedd8∼NAE thioester conjugate immobilized to the streptavidin plate by a mouse anti-HA antibody and an anti-mouse antibody conjugated to horse radish peroxidase (HRP). (b) Results of the ELISA assay with 10× dilution of HA-Nedd8 across the plate. 5 µM Nedd8 was added to the wells in the first column of the streptavidin plate. In the control reactions, either PCP-NAE was not immobilized to the plate or ATP was not added to the reaction.

We cloned the peptidyl carrier protein (PCP) domain as a fusion to the N-terminus of APPBP1, coexpressed the fusion protein with the Uba3 subunit, and purified the PCP-APPBP1-Uba3 heterodimer as the functional NAE following a reported procedure [25]. To verify the activity of PCP tagged NAE, we labeled the PCP domain of the fusion protein with biotin by Sfp phosphopantetheinyl transferase that conjugated the biotin-phosphopantetheinyl group derived from biotin-CoA to the Ser residue of PCP [26]. The biotin-labeled PCP-NAE fusion was immobilized in 96-well plates coated with streptavidin (Figure 2a). Nedd8 with an N-terminal HA tag was added to the plate with ATP and the formation of Nedd8∼NAE conjugate was detected with a mouse anti-HA antibody and an anti-mouse IgG antibody linked to horse radish peroxidase (HRP) (Figure 2a). We observed significant immobilization of HA-Nedd8 in NAE coated wells in the presence of ATP. In contrast, only low levels of HA-Nedd8 were bound to the plate when either NAE or ATP was excluded from the reaction (Figure 2b). These results suggest that PCP tagged NAE immobilized on the streptavidin plate retained catalytic activity with Nedd8 and could be used for the selection of the UB library based on the catalytic formation of UB∼NAE conjugates (Figure 1b).

We next carried out phage selection of a UB library that was constructed in a previous work with randomized residues replacing ^71^LRLRG^75^ at the UB C-terminus [27]. Selection of the phage library was based on the formation of thioester conjugates between phage displayed UB variants and NAE immobilized on the streptavidin plate (Figure 1b). Control reactions were set up in parallel in which either NAE was not immobilized on the plate or ATP was excluded from the reaction mixture. Phage bound to the plate were eluted by DTT and amplified for further rounds of selection. The amounts of phage input, NAE immobilization and reaction time were reduced through iterative rounds of selection to specifically enrich phage displaying UB that were reactive with NAE. There was a steady increase in phage enrichment from the selection over the controls as the selection conditions became more stringent (Table 1). The amount of phage eluted from the fifth round of selection was 1,500-fold and 120-fold higher than the controls missing either NAE or ATP, respectively, suggesting that a substantial fraction of the selected phage pool displayed UB variants that were catalytically active with NAE.

**Table 1:** Enrichment of UB variants reactive with NAE over iterative rounds of phage selection.

Round of Phage Selection	Concentration of input phage (cfu/µL)	Amount of NAEcoated in eachwell (pmol)	Reaction time(min)	Ratio of phage outputSelection/Control excluding NAE	Ratio of phage outputSelection/Control excluding ATP
1	1×10^9^	100	60	40	20
2	8×10^8^	50	30	100	40
3	6×10^8^	25	15	320	65
4	3×10^8^	10	10	800	70
5	1×10^8^	5	5	1500	120

**Figure 3:**
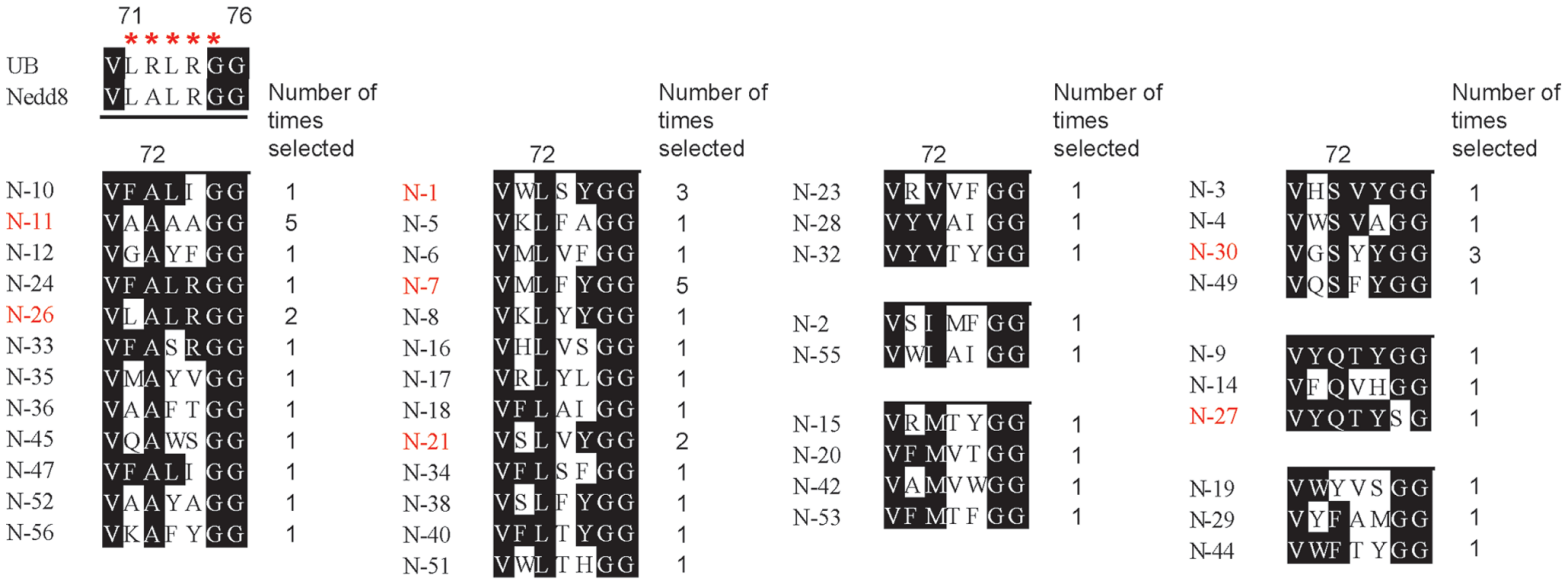
Alignment of the C-terminal sequences of the phage selected UB clones which are reactive with NAE. Sequences are grouped according to the identities of the residues selected at position 72. UB residues 71–75 were randomized in the library and they are designated with the red stars. Names of UB variants that were expressed and further analyzed for reactivity with NAE are shown in red. Numbers of times selected refers to how often a UB clone was identified among the 58 clones sequenced after the fifth round of phage selection.

Sequencing of the UB clones after the fifth round of selection showed a convergence of the UB C-terminal sequences recognized by NAE (Figure 3). The gating residue Arg72 of UB is most commonly replaced by hydrophobic residues with aliphatic side chains including Ala, Leu, Ile, Val, or infrequently with polar residues such as Ser or Gln, or large aromatic side chains such as Phe and Tyr. No positively charged residue such as Arg (native sequence of UB), Lys or His was selected suggesting that NAE rejects positively charged residues at position 72 to avoid activation of the wt UB. Out of 58 clones sequenced, only one clone (N27) has a Ser replacing Gly75 suggesting that the Gly-Gly motif at the C-terminal end of UB is important for reactivity with NAE. Compared to residues 72 and 75, residues selected at positions 71, 73 and 74 can be more diverse with a significant preference for aromatic side chains replacing the native residues Leu71, Leu73 and Arg74 at the C-termini of wt Nedd8 and wt UB (Figure 3).

UB clone N26 with the single Arg72Ala mutation appeared twice among the 58 clones sequenced (Figure 3). This clone has the same C-terminal sequence as Nedd8. This suggests that other UB clones in the selected pool with substantial sequence variations can compete with the native C-terminal sequence of Nedd8 for NAE activation. Interestingly clone N11 with a C-terminal sequence ^71^AAAAGG^76^ was identified five times among all selected clones (Figure 3). Similar to N11, N52 with the C-terminal sequence of ^71^AAYAGG^76^ was also selected suggesting that Ala replacement of Leu and Arg at positions 71, 73 and 74 can still lead to NAE activation of the UB mutant. Thus, the native residues at these positions in UB or NAE should only have limited contributions to Nedd8 recognition by NAE.

### Modeling the Interaction between NAE and the C-terminal Sequences of UB Selected by Phage Display

**Figure 4:**
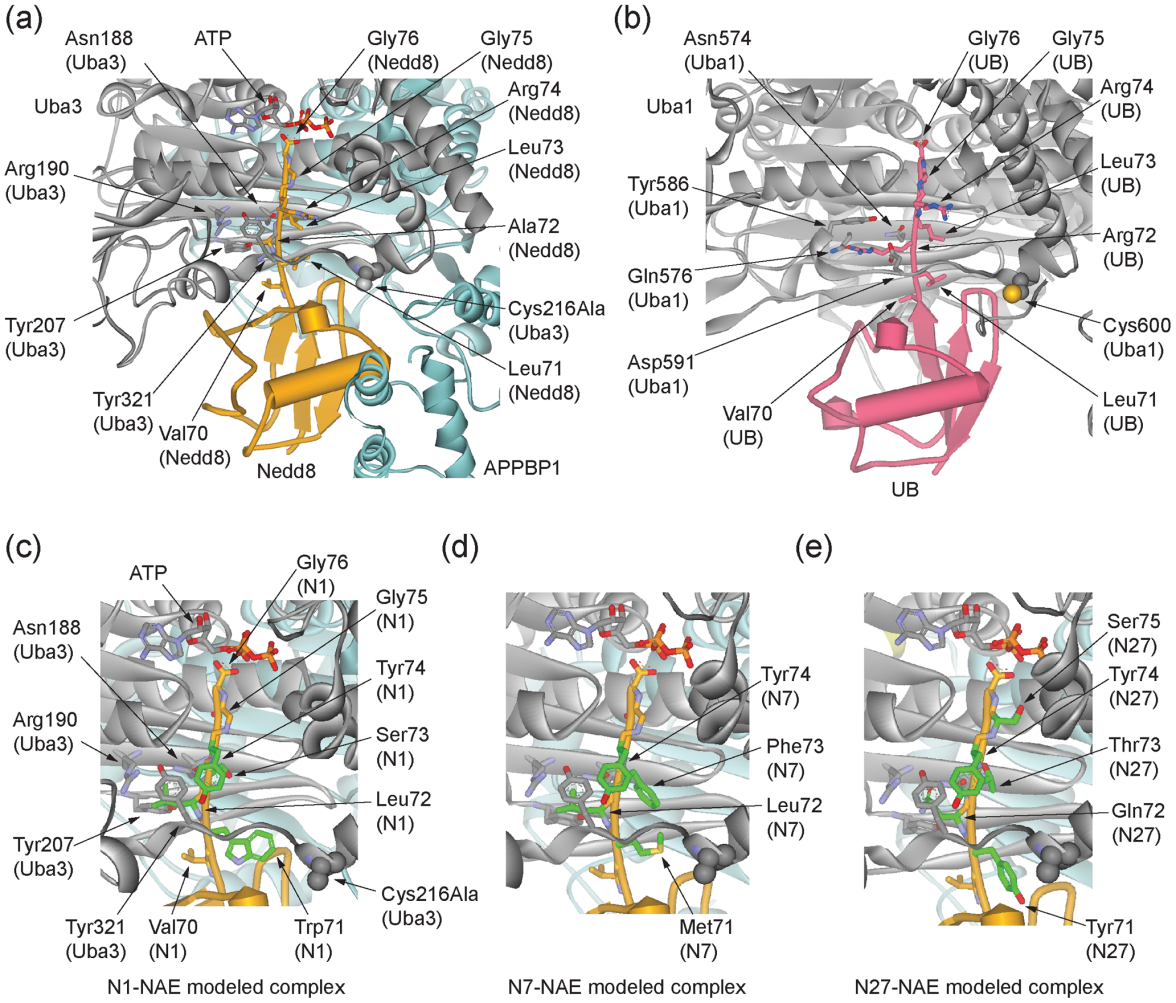
Structural analysis of Nedd8-NAE, UB-UAE, and UB variants -NAE interactions. (a) Binding of Nedd8 to NAE in the Nedd8-NAE-ATP complex (PDB ID 1R4N) [16]. The Uba3 subunit of NAE is colored in grey, the APPBP3 subunit in cyan, and the Nedd8 protein in orange. Key residues of Uba3 for binding to Ala72 of Nedd8, and the Nedd8 C-terminal residues ^71^LALRGG^76^ are shown in stick models. Cys216, the catalytic Cys residue for thioester bonding with Nedd8, was mutated to Ala in the complex and the Ala residue is shown in a CPK model. (b) Binding of UB to UAE (PDB ID 3CMM) [20]. Uba1, the yeast UAE, is colored in grey and the UB is in red. Key residues of Uba1 for binding to Arg72 of UB and UB C-terminal residues ^71^LRLRGG^76^ are also shown in stick models. Cys600, the catalytic Cys of Uba1, is shown in a CPK model. (c)-(e) Modeled structures of Nedd8-NAE complexes with the C-terminal sequences of wt Nedd8 replaced by the sequences of UB variants N1, N7 and N27 from phage selection. (c) Nedd8 with the C-terminal sequence of N1 (^71^WLSYGG^76^ ) bound to NAE. (d) Nedd8 with the C-terminal sequence of N7 (^71^MLFYGG^76^) bound to NAE. (e) Nedd8 with the C-terminal sequence of N27 (^71^YQTYSG^76^) bound to NAE.

A comparison of the crystal structures of NAE and UAE in complex with Nedd8 and UB, respectively, showed that Nedd8 and UB bind to the E1 enzymes in a similar mode (Figure 4a and 4b) [16], [20]. The C-terminal peptides of both Nedd8 and UB adopt an extended conformation so that the terminal Gly76 can approach the ATP molecule bound at the bottom of the adenylation domain of the E1 enzymes. The C-terminal peptides of Nedd8 and UB are also wrapped around by a crossover loop of their cognate E1 enzymes to guide their entrance into the ATP binding pocket. The side chains of Ala72 of Nedd8 and Arg72 of UB point into the same direction in the complex structure with the E1s. Ala72 of Nedd8 is docked in a well-defined binding pocket of limited space assembled by Asn188, Arg190, Tyr207 and Tyr321 of the Uba3 subunit of NAE. Similarly Uba1 residues Asn574, Gln576, Tyr586, and Asp591 constitute a larger pocket for specific recognition of Arg72 of UB. Except for Ala72 of Nedd8 and Arg72 of UB, other residues including Leu71, Leu73 and Arg74 at the C-termini of Nedd8 and UB are identical, with Leu71 and Leu73 pointing to the opposite side of NAE and UAE while Arg74 is oriented in a nearly perpendicular direction to that of Ala72 of Nedd8 or Arg72 of UB (Figure 4a and 4b). In case of all three residues (71, 73 and 74), there is significantly more open space around their side chains compared to the residue at position 72. This might explain the enrichment of aromatic residues at positions 71, 73 and 74 when the UB library was selected with NAE in this study and selected with UAE in an earlier study [27].

To further characterize the recognition of the C-terminal sequences of UB variants by NAE, we transplanted the C-terminal peptides of UB variants N1, N7 and N27 onto the Nedd8 scaffold and modeled the binding of the Nedd8 mutants with NAE based on the crystal structure of Nedd8-NAE-ATP complex (PDB ID 1R4N) [16]. The modeled structures showed that NAE can accommodate larger side chains such as Met, Phe, Tyr and Trp at positions 71, 73 and 74 at the C-terminus of Nedd8 (Figure 4c–e). The binding pocket of NAE for the wt Ala72 of Nedd8 is relatively limited in size, so Leu72 in N1 and N7 and Gln72 in N27 seem at first ill-suited to contribute to positive interactions with the NAE residues. However, a more detailed analysis reveals that both Leu72 and Gln72 can be accommodated by the NAE binding pocket after subtle adjustment of their side chain conformations to avoid unfavorable contacts with nearby NAE residues. A crystal structure of the Ala72Gln mutant of Nedd8 in complex with NAE already demonstrated that Gln72 of Nedd8 can fit into the binding pocket of NAE for Ala72 recognition and engage in hydrogen bonding interactions with Arg190 of the Uba3 subunit of NAE [23]. It has been reported that the Arg72Leu mutant of UB can be activated by NAE with a K_m_ of 20 µM, more than 20-fold higher than the K_m_ of wt Nedd8 with NAE (0.95 µM) [15]. The Ala72Gln mutant of Nedd8 was measured to have a K_d_ of 8.75 µM with NAE, 27-fold higher than the K_d_ of wt Nedd8 with NAE (0.333 µM) [23]. These results match the modeling studies and suggest that NAE can still accommodate either Leu or Gln at position 72 of UB or Nedd8 despite the fact that the native residue being recognized by NAE at the same position is the much smaller Ala.

### Reactivity of the Phage Selected UB Mutants with NAE

**Figure 5:**
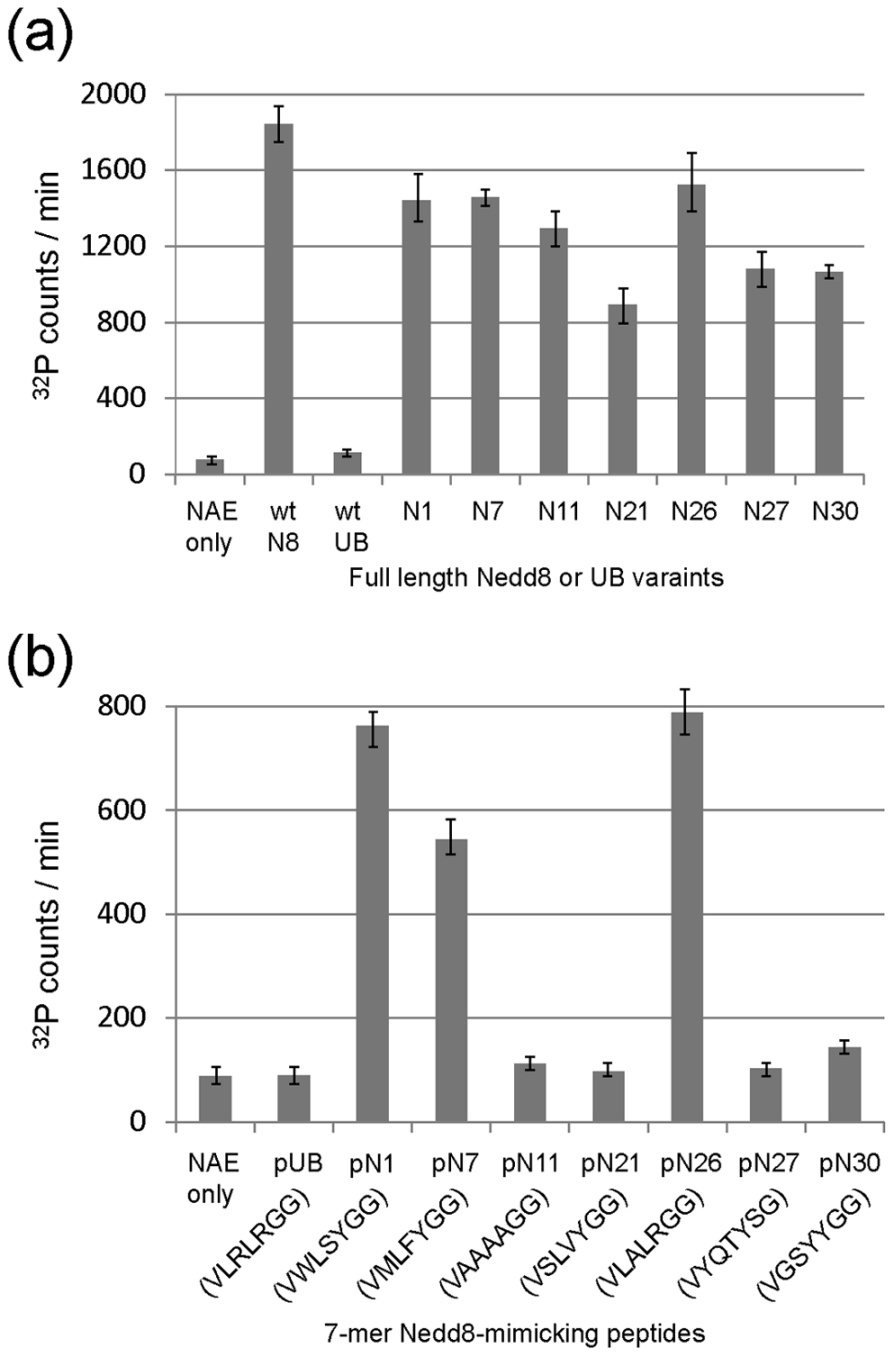
The activities of UB variants and the corresponding C-terminal peptides with NAE measured by the ATP-PP_i_ exchange assay. (a) Activities of full-length Nedd8 or UB variants with NAE. (B) Activities of the heptameric Nedd8-mimicking peptides (sequences indicated in single letter code) derived from the UB variants with NAE.

To assay the activities of NAE with the UB mutants identified by phage display, we subcloned the UB mutants N1, N7, N11, N21, N26, and N30 that appeared multiple times among the sequenced clones (Figure 3). We also expressed the UB mutant N27 since it was selected with a Ser residue replacing Gly75. The UB mutants were cloned into a pET vector and were expressed with an N-terminal HA tag to facilitate the detection of UB conjugates with NAE and Ubc12. We used the ATP/PP_i_ exchange assay [28], [29] to measure the kinetics of NAE activation of UB mutants. In this assay, NAE catalyzes the condensation of UB with ATP to form a UB-AMP conjugate coupled to the release of PP_i_. Externally added PP_i_ labeled with the radioactive isotope ^32^P sets the reaction in reverse thus incorporating ^32^PP_i_ into ATP. Radioactive ATP formed in the reaction is captured by charcoal and quantified. The rate of ^32^PP_i_ exchange into ATP reflects the reactivities of NAE with the UB variants to form UB-AMP intermediates. The ATP/PP_i_ exchange assay confirmed that NAE only has limited activity with wt UB with a k_cat_/K_1/2_ of 0.012 µM^−1^min^−1^ that is more than 3,500 fold lower than the k_cat_/K_1/2_ of wt Nedd8 with NAE (43 µM^−1^min^−1^) (Figure 5a and Table 2). We also found that UB cannot saturate NAE at concentrations as high as 200 µM while Nedd8 has a K_1/2_ of 1.3 µM with NAE, suggesting that binding of wt UB to NAE is significantly hampered thus increasing the specificity of NAE towards Nedd8. In contrast to wt UB, UB variants from phage selection can be activated by NAE at a level similar to that of wt Nedd8 (Figure 5a and Table 2). N1 and N7 are ending with the C-terminal sequences ^71^WLSYGG^76^ and ^71^MLFYGG^76^, respectively, with aromatic residues at positions 71, 73 and 74 flanking Leu72. The Arg72Leu single mutant of UB was previously found to be activated by NAE [15] and we observed that the k_cat_/K_1/2_ of N1 and N7 with NAE are both 16 µM^−1^min^−1^, just less than half of that of wt Nedd8 (Table 2). N1 and N7 have K_1/2_ values of 1.8 and 2.0 µM, matching the K_1/2_ of wt Nedd8 (1.3 µM), or N26 (1.6 µM) with the wt C-terminal sequence of Nedd8 on a UB scaffold. These results confirm that large aromatic side chains at position 71, 73 and 74 of Nedd8 can be well accommodated by NAE.

**Table 2:** Kinetic characterization of the UB variants and Nedd8-mimicking peptides in the ATP/PPi exchange reactions catalyzed by NAE.

	K_m_ (µM)	k_cat_ (min^−1^)	k_cat_/K_m_ (µM^−1^min^−1^)
Full-length proteins			
Nedd8 (^71^LALRGG^76^)	1.3±0.2	55±12	43
wt UB (^71^LRLRGG^76^)	ND	ND	0.012
UB variants from phage selection			
N1 (^71^WLSYGG^76^)	1.8±0.3	30±2	16
N7 (^71^MLFYGG^76^)	2.0±0.2	32±5	16
N11 (^71^AAAAGG^76^)	2.2±0.3	34±6	16
N27 (^71^YQTYSG^76^)	2.8±0.8	26±3	9.0
N26 (^71^LALRGG^76^)	1.6±0.2	52±16	32
Nedd8 mimicking peptides			
pN1 (^70^VWLSYGG^76^)	130±41	24±4	0.18
pN7 (^70^VMLFYGG^76^)	155±50	24±6	0.16
pN26 (^70^VLALRGG^76^)	88±25	26±7	0.29

ND, not determined because of low activity.

N26 is the Arg72Ala mutant of UB and was previously found to be active with NAE by site directed mutagenesis [16]. We found that the activity of N26 with NAE (32 µM^−1^min^−1^) is approaching that of Nedd8 (43 µM^−1^min^−1^), confirming the important role of UB residue Arg72 to prevent UB from cross reacting with NAE (Table 2). Interestingly, N11 with the UB C-terminal sequence ^71^LRLR^74^ all replaced by Ala, was also selected by phage display. N11 can be efficiently activated by NAE with a k_cat_/K_1/2_ of 16 µM^−1^min^−1^, the same value obtained for NAE activation of the N1 and N7 that have bulky aromatic residues Phe, Tyr and Trp at positions 71, 73 and 74. N11 also has a K_1/2_ of 2.2 µM that is slightly higher than that of N1, N7 and N26 from phage selection or the wt Nedd8 (Table 2). These results suggest that NAE mainly recognizes residue 72 at the UB C-terminus and has substantial promiscuity towards residues at positions 71, 73 and 74. We further found that N27 with Gly75 mutated to Ser can also be activated by NAE with a K_1/2_ of 2.8 µM, close to the UB variants with an intact Gly-Gly motif at the C-terminus.

**Figure 6:**
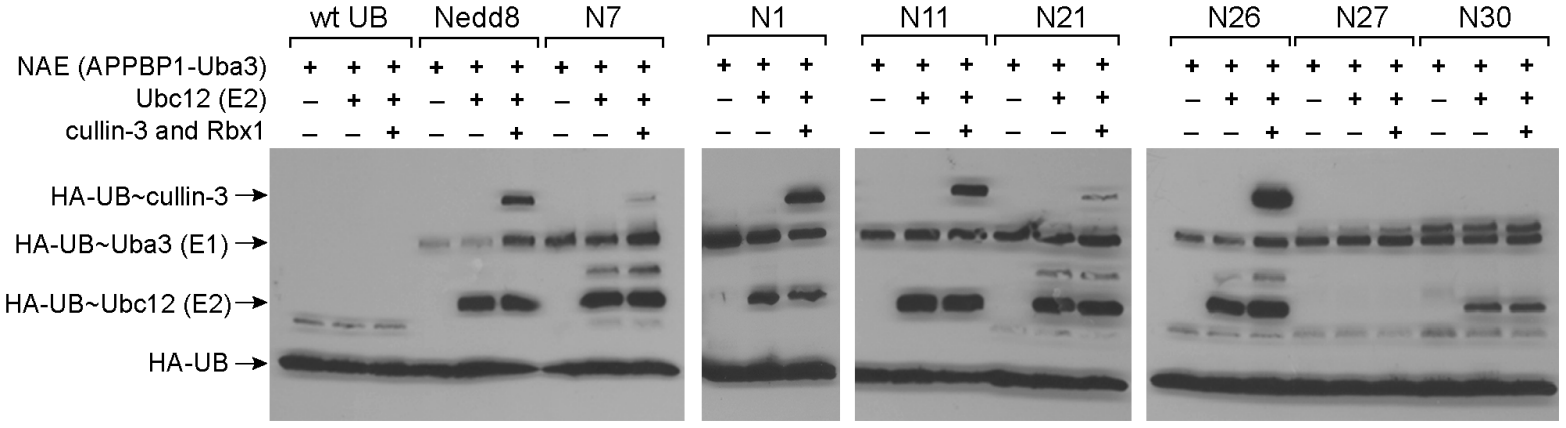
Transfer of phage selected UB variants to NAE (E1), Ubc12 (E2), and cullin-3. HA-tagged wt UB, wt Nedd8 and UB variants were used in the assay. In the control reaction, either Ubc12 or cullin-3 in complex with Rbx1 was missing. The blots were probed with a mouse anti-HA antibody and a goat anti-mouse IgG antibody linked to HRP.

Next we assayed the formation of thioester conjugates between UB variants and NAE and the subsequent transfer of UB variants to the E2 enzyme Ubc12 and the cullin subunit. As shown in Figure 6, all UB variants from phage selection can form UB∼NAE conjugates at levels comparable to wt Nedd8, while wt UB could not form conjugates with NAE at an observable level. Among the phage selected UB mutants, several can also be transferred from NAE to Ubc12 including N1, N7, N11, N21, N26, and N30. N11 has the C-terminal sequence ^71^AAAAGG^76^_,_ thus the Ala mutations at position 71, 73 and 74 do not significantly affect Nedd8 transfer to Ubc12. N26 has the native C-terminal sequence of Nedd8 and it efficiently forms UB∼Ubc12 conjugates. N1, N11 and N26 can give substantial cullin modification at a level similar or exceeding that of wt Nedd8. In contrast N7, N21, and N30, despite them being loaded on Ubc12 by NAE, cannot be transferred to cullin at significant levels. These UB variants have aromatic residues at 73 and 74 that may affect UB transfer to cullin. N27 is a UB variant that can only be conjugated to NAE but does not form a UB∼Ubc12 conjugate. N27 has the C-terminal sequence of ^71^YQTYSG^76^ with Gly75 in the double Gly motif of UB being replaced by a Ser residue. This mutation apparently leads to inefficient transfer of the UB variant from NAE to Ubc12.

### Transfer of Phage Selected C-terminal Peptides of UB to NAE and Ubc12

We previously found that the 7-mer peptides derived from the C-termini of UB variants from phage selection with UAE can form thioester conjugates with UAE and the E2 enzymes of the UB transfer cascade [30]. We referred to these peptides as “UB-mimicking peptides” and we further showed that once the peptides are loaded on UAE and the E2 enzymes, the cascade enzymes are blocked from transferring UB to E3 for protein ubiquitination. We were thus interested in assaying if the C-terminal peptides of the UB variants selected by NAE can form thioester conjugates with NAE and Ubc12. We synthesized peptides corresponding to the last seven residues of the UB variants N1, N7, N11, N21, N26, N27, and N30 which are referred to as pN1, pN7, pN11 and so on. The full-length proteins of these UB variants all showed similar activities with NAE as wt Nedd8 in the ATP-PP_i_ exchange reaction (Figure 5a and Table 2). We assayed the ATP-PP_i_ exchange activity of the peptides with NAE and found that peptides pN1, pN7 and pN26, derived from the C-terminal sequences of UB variants N1, N7 and N26, can be activated at a level significantly higher than the background with no peptide added (Figure 5b). Kinetic analysis of the three peptides suggested that the peptides have similar k_cat_ values in the range of 23–26 min^−1^, comparable to that of the wt Nedd8 protein with a k_cat_ of 55 min^−1^ (Table 2). In contrast the peptides have K_1/2_ between 88 – 155 µM, which are 100-fold higher than full-length Nedd8 suggesting that other interfaces between UB and NAE significantly contribute to NAE recognition besides the C-terminal peptides of the UB variants. Peptide pN26 has the native C-terminal sequence of wt Nedd8, documenting that the C-terminal peptide of Nedd8 alone has substantial activity with NAE.

**Figure 7:**
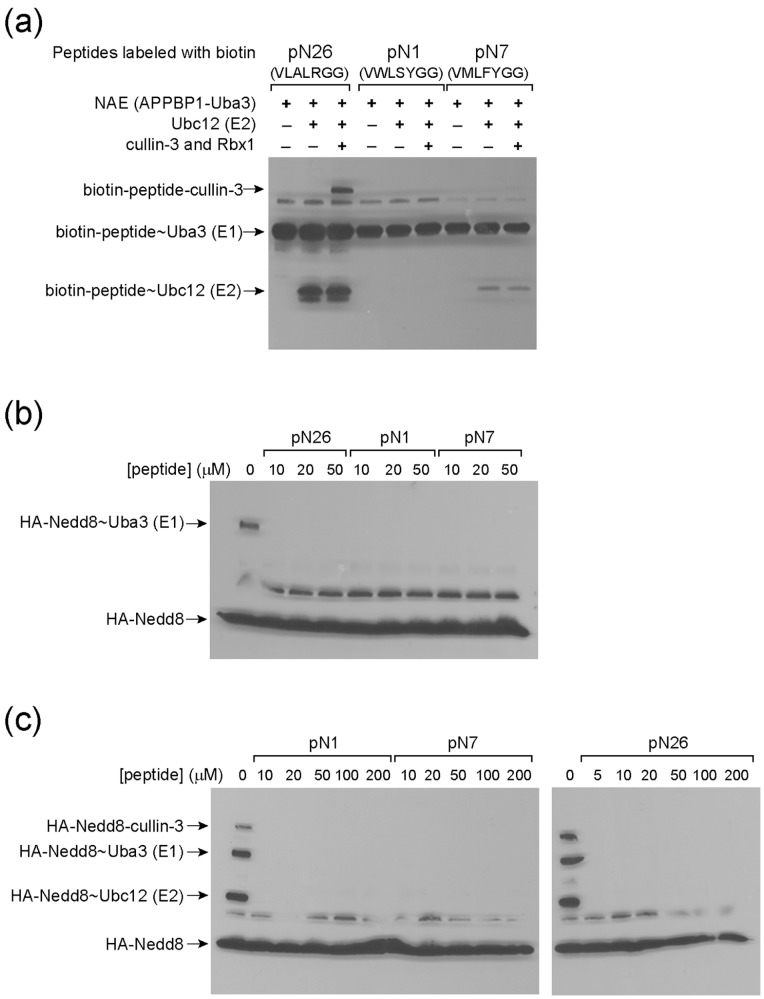
Transfer of Nedd8-mimicking peptides to NAE, Ubc12 and cullin-3, and inhibition of Nedd8 transfer through the cascade enzymes. (a) Transfer of peptides pN1, pN7 and pN26 to NAE, Ubc12 and cullin 3. The peptides were conjugated to biotin at their N-termini. The Uba3 subunit of NAE formed the thioester conjugate with the peptides. The Western blot was probed with streptavidin conjugated to HRP. (b) Inhibiting the formation of Nedd8∼NAE conjugates by the Nedd8-mimicking peptides. (c) Inhibiting Nedd8 transfer to Ubc12 and cullin-3 proteins by the peptides.

We next conjugated biotin to the N-termini of the peptides pN1, pN7, and pN26 and found that all three peptides can form thioester conjugates with NAE based on the Western blot of the transfer reaction probed with streptavidin-HRP conjugate (Figure 7a). We thus refer to these peptides as “Nedd8-mimicking peptides” since they can be activated by NAE and form thioester conjugates with NAE as the wt Nedd8 protein. However, only peptide pN26, with its sequence matching the C-terminus of native Nedd8, showed a strong activity for the formation of peptide∼Ubc12 conjugates and the peptide can be further transferred to cullin 3 (Figure 7a). In contrast peptide pN7 only has low activity for transferring to Ubc12 while pN1 showed no observable formation of peptide∼Ubc12 conjugates, though both peptides can be loaded on NAE at a similar level as pN26 (Figure 7a). These results indicate that only the native Nedd8 C-terminus present in pN26 retains the ability to pass through the NAE-Ubc12 cascade for cullin modification. The non-native residues in pN1 and p27 may block the peptides from transferring to Ubc12 and cullin although they do not interfere with NAE recognition.

### Inhibiting the NAE-Ubc12 Cascade by the Nedd8 Mimicking Peptides

We rationalized that if the Nedd8-mimicking peptides can be loaded on NAE and Ubc12, they should block Nedd8 transfer through the cascade since the peptides occupy the catalytic Cys residues of NAE and Ubc12 to prevent Nedd8 conjugation. These peptides can serve as road blocks to stop Nedd8 transfer through the cascade. Indeed after we incubated the peptides with NAE to allow the charging of NAE with the peptides, the addition of full-length Nedd8 failed to yield any Nedd8∼NAE conjugate (Figure 7b). Furthermore when the Nedd8-mimicking peptides pN1, pN7 and pN26 were incubated with NAE, Ubc12 and cullin to allow the preloading of the peptides to the enzymes of the Nedd8 transferring cascade, the chase of the loading reaction with full-length Nedd8 did not produce any Nedd8 conjugates with NAE, Ubc12 or cullin (Figure 7c). These results demonstrate that the Nedd8-mimicking peptides can be used as inhibitors to block cullin neddylation.

## Discussion

### UB C-terminal Residues for NAE and UAE Recognition

In this study we carried out phage selection of a UB library with NAE to reveal its selectivity for the UB C-terminal sequences. Since we previously used UAE to select for reactive UB variants from the same library [27], we can now compare the profiles of UB C-terminal sequences that are recognized by NAE and UAE, respectively. The sequence alignment of UB variants from UAE selection showed that all selected UB clones retained Arg72 in the wt UB clearly indicating that the positively charged Arg side chain at this position is essential for UAE recognition [27]. In contrast UB variants from NAE selection have Arg72 of wt UB replaced by hydrophobic side chains of variable sizes such as Ala, Val, Leu, Ile, Phe, Tyr, and Met or, more infrequently, polar side chains Ser or Gln (Figure 3). These results match earlier observations that residue 72 provides a gating mechanism for UAE and NAE to differentiate between UB and Nedd8, with UAE recognizing Arg and NAE recognizing Ala or Leu [15], [16], [23]. The phage selections with UAE and NAE further reveal that UAE has a strict selectivity for Arg72 of UB and NAE selects against Arg and other positively charged residues such as Lys or His at the same position, although NAE readily tolerates variations at position 72 among hydrophobic or even uncharged polar residues (Figure 3).

Based on the crystal structure of UB in complex with the yeast UAE, Uba1, Arg72 of UB is bound to the adenylation domain of Uba1 in a pocket surrounded by Asn574, Gln576, Tyr586 and Asp591 (Figure 4b) [20]. The specificity of Arg72 of UB may arise from the electrostatic and hydrogen bonding interactions between the guanidino group of Arg72 and Uba1 residues Gln576 and Asp591. The two corresponding residues in NAE for interaction with Ala72 of Nedd8 are Arg190 and Tyr207 in the Uba3 subunit. Because of the small size of the methyl side chain of Ala72, Arg190 of Uba3 has no direct contact with Ala72 but rather its role has been assigned to reject the binding of Arg72 of UB [16], [23]. Arg190 can have productive hydrogen bonding interactions with a Gln residue at position 72 of Nedd8 as suggested by the crystal structure of NAE in complex with the Ala72Gln mutant of Nedd8 [23] and our model building studies on N27-NAE complex (Figure 4e). Phage selection of the C-terminal UB library has identified mutants with the Arg72Gln mutation on UB to enable its activation by NAE (N9, N14 and N27 in Figure 3). Presumably the Gln72 residue on the UB scaffold can engage with Arg190 of NAE just as the Ala72Gln mutant of Nedd8 [23]. The Ala72 binding pocket of NAE seems to be more tolerable of changes than the Arg72 binding pocket of UAE since residues of variable sizes (Ala, Val, Leu, Ile, Phe, Tyr and Met) and polarities (Ser and Gln) at position 72 of the UB variants are all compatible with NAE activation.

Phage profiling with NAE and UAE also shows that both E1 enzymes have a relaxed selectivity towards residues 71, 73 and 74 at the UB C-terminus. Aromatic residues Phe, Tyr and Trp have a high frequency to be selected at these positions by both E1s. This indicates that there should be additional space in both types of E1 to accommodate the bulkier residues. The crystal structures of both NAE and UAE show that the C-terminal peptides of UB and Nedd8 project away from the core of the β-grasp fold in order to approach the ATP cofactor bound at the bottom of the adenylation domains (Figure 4a and 4b) [20], [22]. The binding interface of the E1 adenylation domains with the C-terminal peptides of UB or Nedd8 is like a funnel that gets narrow at the bottom to fit the Gly-Gly motif at the very end of the UB or Nedd8 C-termini. Leu71, Leu73 and Arg74 of either UB or Nedd8 only have few interactions with the E1 enzymes. The replacement of these residues with aromatic side chains may help to expand the binding interface of the UB variants with the E1s. Interestingly N11, the UB variant with the C-terminal sequence of ^71^AAAAGG^76^ has also been selected from the phage library by NAE. This mutant is equal to the triple Ala mutant of N26, the UB variant with the native ending of Nedd8 (Figure 3). Since N11 is activated by NAE at similar efficiencies as N26 and wt Nedd8 both ending with ^71^LALRGG^76^ (Table 2), the size and identities of residues at positions 71, 73 and 74 of UB may not be important factors in NAE recognition.

We also found that both UAE and NAE have a strong preference for Gly75 so as to preserve the Gly-Gly motif at the end of UB (Figure 3). Still Gly75 can be replaced by Ser for NAE recognition, and by Ser, Asp, Asn and Gln for UAE recognition [27].

### Variations in the C-terminal Sequence of UB Affect UB Transfer to Ubc12 and Cullin

We found in this study that C-terminal variations in UB may affect its transfer through the NAE-Ubc12-cullin cascade. The UB variant N26 ending with the native Nedd8 sequence ^71^LALRGG^76^ can function as the wt Nedd8 for thioester formation with NAE and Ubc12 and subsequent transfer to cullin-3 for protein modification (Figure 6). Similarly N11, ending with ^71^AAAAGG^76^, can also be transferred through the NAE-Ubc12 cascade to modify cullin at a similar level as N26 and the wt Nedd8 (Figure 6). UB variants N7, N21 and N30 have bulky aromatic residues replacing native UB residues at positions 71, 73 and 74. These UB variants are activated by NAE with similar activities as that of N26 and wt Nedd8 based on the ATP-PP_i_ assay (Table 2). At a level similar to N26 and wt Nedd8, they can also form thioester conjugates with NAE and be transferred to Ubc12 to form UB∼E2 conjugates, however, their activity to be further transferred to cullin has been significantly reduced (Figure 6). N27 with the C-terminal sequence of ^71^YQTYSG^76^ has a normal level of NAE activation and thioester formation with NAE, but its transfer to Ubc12 and cullin is blocked, most likely due to the Ser residue replacing Gly75 that alters the Gly-Gly motif at the end of UB and Nedd8 (Figure 6). These results suggest that the variation of UB C-terminal sequence may significantly affect UB transfer through the Nedd8 cascade.

We previously observed that C-terminal variations of UB may affect the ability of the UB mutants to pass through the UB transfer cascade [27]. While UAE-selected UB variants with the native Arg72 and non-native residues at 71, 73, 74 and 75 in the C-terminal sequence ^71^LRLRGG^76^ can all form thioester conjugates with UAE, UB variants with Gly75 replaced by Asp, Asn, and Gln that are significantly larger than Gly have reduced or defective transfer to the E2 enzymes such as UbcH7, however, UB variants with Ser replacing Gly75 do not affect UB transfer to E2. We further found that non-native residues replacing Arg74 and Gly75 block UB transfer from E2 to E3s of either HECT type for the formation of UB∼HECT thioester conjugate or RING/U-box types for self-ubiquitination. These results suggest that the C-terminal sequences of UB and UB variants are closely monitored by the cascade enzymes during their passage through the UB or the Nedd8 cascade.

### Inhibition of Nedd8 Transfer through the NAE-Ubc12 Cascade by the Nedd8-mimicking Peptides

We also found that 7-mer peptides corresponding to the C-terminal sequence of NAE selected UB mutants are reactive with NAE in the ATP-PP_i_ assay (Table 2). These peptides can form thioester conjugates with NAE (Figure 7a). Peptide pN26 derived from N26 with the native C-terminal sequence of Nedd8 can be further transferred to Ubc12 and cullin to form covalent conjugates (Figure 7a). Peptide pN11 with the sequence ^71^VAAAAGG^76^ is not reactive with NAE (Figure 5b), although the corresponding UB variant N11 can be activated by NAE with a similar efficiency as the wt Nedd8 or N26 (Figure 5a and Table 2) and be transferred through the NAE-Ubc12 cascade for cullin modification (Figure 6). This suggests that the N11 variants of UB should rely on interfaces other than its C-terminal peptide for binding to NAE. The pN11 peptide itself cannot be recognized by NAE for the activation reaction.

Peptides pN1 (^71^VWLSYGG^76^) and pN7 (^71^VMLFYGG^76^) derived from UB variants N1 and N7 were found to be activated by NAE at a similar efficiency as the pN26 peptide (Table 2). They also form peptide∼NAE conjugates at a similar level as pN26 (Figure 7a). However, these peptides are less efficient for transferring to Ubc12 and none of them can form peptide-cullin conjugates at a detectable level. Both peptides have bulky aromatic substitutions of the wt residues at positions 71, 73 and 74. These results again suggest that the C-terminal sequence of UB would greatly affect the activity of peptide transfer to Ubc12 and cullin.

Finally we showed that NAE loaded with the Nedd8-mimicking peptides pN1, pN7 and pN26 is prevented from transferring Nedd8 through the Nedd8-Ubc12 cascade for cullin modification (Figure 7b and 7c). Since these peptides rely on NAE catalyzed activation and peptide∼NAE thioester formation to inhibit cullin modification, they should be classified as mechanism-based inhibitors of the Nedd8 transfer cascade. Recently the compound MLN4924 was developed by Millennium as a potent inhibitor of NAE [31], [32]. MLN40924 binds to the nucleotide binding pocket of NAE as an AMP analog with a sulfamate group replacing the phosphate moiety of AMP. The amino group of the sulfamate attacks the thioester linkage between Nedd8 and the catalytic Cys residue of NAE leading to the formation of a Nedd8-MLN4924 adduct that binds to the adenylation domain of NAE with high affinity. The Nedd8 mimicking peptides block the catalytic Cys residues of NAE and prevent the loading of NAE with full-length Nedd8. Peptide pN26 with the native C-terminal sequence of Nedd8 can also be transferred to Ubc12 to block Nedd8 conjugation to E2. It can be further transferred to cullin to block Nedd8 modification of cullin (Figure 7a). Peptides pN1 and pN7, however, are stalled at the stage of forming the peptide∼NAE conjugate and are significantly less active in further transfer to Ubc12 or cullin. Thus, these peptides can function as road blocks of Nedd8 transfer at different stages of the NAE-Ubc12 cascade. They provide a mechanism for inhibiting cullin modification that is distinct from MLN4924. It will be of interest to further probe the effects of the Nedd8-mimicking peptides on the activity of cullin-RING ligase in the cell.

## Materials and Methods

### Molecular Cloning

Plasmid pGEX-GST-APPBP1-Uba3 was constructed for the coexpression of the APPBP1 and Uba3 subunits of NAE in the *E. coli* cell with a glutathione S-transferase (GST) tag fused to the N-terminus of APPBP1 [25]. For the expression of PCP-NAE fusion, the gene fragment encoding GST in pGEX-GST-APPBP1-Uba3 between the NheI and NotI restrictions sites was replaced with the gene of the peptidyl carrier protein (PCP) domain of GrsA [26] with an N-terminal 6×His tag to generate the plasmid pGEX-PCP-APPBP1-Uba3. This plasmid was used for the coexpression of the PCP-APPBP1 fusion and Uba3 to assemble PCP-NAE. For the expression of HA-Nedd8 with an N-terminal HA tag, the Nedd8 gene was amplified by polymerase chain reaction (PCR) from pGEX-NEDD8 [25] and cloned into pET28a between the NheI and NotI restriction sites. The pET-HA-Nedd8 plasmid expressed HA-Nedd8 fusion with an N-terminal 6×His tag.

### Protein Expression and Purification

For the expression of proteins with a 6×His tag from the pET or pGEX vectors, the plasmids were transformed into the BL21(DE3)pLysS chemical competent cells (Invitrogen) and plated on LB-agar plates with appropriate antibiotics. Protein expression and purification followed the protocol provided by the vendors of the pET expression system (Novagen) and the Ni-NTA agarose resin (Qiagen). Typically the cells were grown in 1 L Lysogeny Broth (LB) supplemented with 100 µg/mL ampicillin to an OD around 0.6–0.8 at 37°C. The LB culture was then induced by adding IPTG to a final concentration of 1 mM and incubating with continuous shaking at 15°C overnight. Cells were subsequently collected by centrifugation at 5,000 rpm for 10 min, resuspended in lysis buffer (50 mM Tris base, 500 mM NaCl, 5 mM imidazole, pH 8.0) and lysed with French press (Thermo Scientific). The resulting crude suspension was centrifuged at 12,000 rpm to remove the pelleted cell debris from protein lysate. The lysate supernatant was mixed with 1 mL of Ni-NTA agarose (Qiagen) and rocked gently at 4°C for 2 hours. The slurry was next transferred to a gravity column, washed once with 15 mL lysis buffer, twice with 15 mL wash buffer (50 mM Tris base, 500 mM NaCl, 20 mM imidazole, pH 8.0) and eluted with 5 mL elution buffer (50 mM Tris base, 500 mM NaCl, 250 mM imidazole, pH 8.0). Eluted protein was dialyzed overnight at 4°C in a 1 L buffer (25 mM Tris base, 150 mM NaCl, 0.5 mM DTT, pH 8.0), followed by a second dialysis the next day with the same buffer for 3 hours. All purified proteins were assayed by electrophoresis on a 4–15% SDS Tris polyacrylamide gel (PAGE) (Biorad) for the verification of their sizes and purity, and eventually stored in aliquots at −80°C. Ubc12 and the cullin3-Rbx1 complex was expressed and purified as previously reported [33].

### Biotin Conjugation to PCP-NAE Fusion

Biotin labeling of PCP-NAE catalyzed by Sfp phosphopantetheinyl transferase followed a reported protocol [26]. 100 µL labeling reaction was set up containing 5 µM PCP-NAE, 2 µM biotin-CoA, 0.3 µM Sfp in a reaction buffer (50 mM HEPES, 10 mM MgCl_2_, pH 7.5). The reaction was allowed to proceed for 1 hour at 30°C, and then mixed with 100 µL 3% BSA. 100 µL of the reaction mixture was distributed to a 96-well plate coated with streptavidin and allowed to bind to the plate for 1 hour at room temperature. The plate was then washed three times with TBS buffer (20 mM Tris HCl, 150 mM NaCl, pH 7.5) to remove unbound enzymes before the phage selection reaction.

### Enzyme-linked Immunosorbent Assay (ELISA)

The transfer of Nedd8 to biotin labeled PCP-NAE bound to the streptavidin plate was analyzed by ELISA. After the labeling reaction, PCP-NAE attached with biotin was bound to a streptavidin plate and the plate was washed with TBS. 5 µM Nedd8 protein with an N-terminal HA tag was added to the streptavidin plate in the presence of 5 mM ATP in TBS. Control reactions were also set up excluding ATP or using a streptavidin plate without the coating of PCP-NAE. After a 1 hour incubation, the plate was washed and the Nedd8 protein bound to the plate was detected by binding consecutively with a mouse anti-HA antibody (Santa Cruz Biotechnology) (1∶500 dilution in TBS with 3% BSA) and a goat anti-mouse antibody conjugated with HRP (Pierce) (1∶20,000 dilution in TBS with 3% BSA). The peroxidase activity immobilized on the plate was assayed by the addition of a 1∶1 mixture of tetramethylbenzidine (TMB) and hydrogen peroxide solution (Thermo Fisher Scientific). The ELISA plate was incubated at room temperature for 5 minutes before imaging.

### Phage Selection of the UB Library

Construction of the UB library in the pJF3H vector [34], [35] with randomized C-terminal residues and the display of the library on the surface of M13 phage were previously reported [27]. In the first round of phage selection for UB variants reactive with NAE, biotin-labeled PCP-NAE fusion was bound to the streptavidin plate at a level of 100 pmol PCP-NAE in each well of the plate. After washing the plate with TBS, 100 µL phage displaying the UB library was added to each well in the reaction buffer (1.5% BSA, 1 mM ATP, and 50 mM MgCl_2_ in TBS). Approximately 1×10^11^ phage particles were added to each well and a total of 8 wells were used for phage selection. The conjugation reaction of UB with NAE was allowed to proceed for 1 hour at room temperature. The reaction mixture was then removed from the well, and the plate was washed with TBS-T (0.05% (v/v) Tween 20, 0.05% (v/v) Triton X-100 in TBS) 30 times each time with 200 µL TBS-T. The plate was further washed with TBS for 30 times. After washing, phage bound to the plate were eluted by adding 100 µL elution buffer (20 mM dithiothreitol (DTT) in TBS) to each well. After 15-minute incubation, the elution buffer was taken out from the well, combined, and added to 10 mL log phase *E. coli* XL1-blue cells. The cell culture was shaken at 37°C for 1 hour to allow phage infection followed by plating out the cells on LB agar plates supplemented with 2% (w/v) glucose and 100 µg/mL ampicillin. After overnight incubation at 37°C, colonies on the plates were harvested, and the phagemid DNA was extracted with a QIAprep plasmid miniprep kit (Qiagen). The phagemid DNA was then used for the next round of phage amplification and selection. After each round of selection, phage particles eluted from the selection or the control reactions were titered. During iterative rounds of selection, the number of the input phage particles, the amount of PCP-NAE coated on the plate, and the reaction time were decreased to increase the selection stringency. Eventually for the fifth round of selection, 10^10^ phage particles in 100 µL reaction mixture were incubated with 1 pmol NAE coated in a well of the streptavidin plate for 5 minutes at room temperature. After the fifth round of selection, phage clones were sequenced.

### ATP-PP_i_ Exchange Assay

50 µL reactions were set up containing 5 µM wt Nedd8 or UB variants, 0.5 µM NAE, 50 mM Tris-HCl, pH 7.5, 10 mM MgCl_2_, 1 mM ATP and 1 mM sodium [^32^P]pyrophosphate (4.6 Ci/mol). The reaction was incubated for 30 min at room temperature and then quenched by the addition of 0.5 mL slurry of charcoal in 2% trichloroacetic acid. After centrifugation, the charcoal pellet was washed 3 times each time with 1 mL 2% trichloroacetic acid, and eventually resuspended in 0.5 mL water and 3.5 mL Ultima Gold LSC-cocktail (Perkin Elmer). The radioactivity bound to charcoal was determined by liquid scintillation counting. To assay the activities of the Nedd8-mimicking peptides with NAE, 50 µM peptide was used in the reaction.

The kinetics for the activation of wt Nedd8 and UB variants by NAE were characterized based on the ATP-PP_i_ exchange assay. The initial velocity of the ATP-PP_i_ exchange was determined in the presence of 0.05 µM NAE with varying concentrations of wt Nedd8 or UB variants in the range of 0.05 µM to 5 µM. The reaction was incubated at room temperature for 5, 10, 15, 20 and 25 minutes before it was quenched with the addition of 0.5 mL slurry of charcoal in 2% trichloroacetic acid. The radioactivity incorporated into charcoal-bound ATP was measured by liquid scintillation counting. K_1/2_ and k_cat_ values of the ATP-PP_i_ exchange reaction were calculated by fitting the data to the Michaelis-Menten equation with the data analysis software Origin. To measure peptide activation by NAE, 0.1 µM NAE was used in the reaction with the concentration of the peptides varying from 10 µM to 1000 µM.

### Biotin Conjugation of the Peptides

Heptameric peptides with the C-terminal sequences of wt UB, wt Nedd8, and UB variants from phage selection were ordered from EZBiolab (Carmel, Indiana). The peptides were further purified by HPLC to be more than 95% pure. Peptide pN1 (2 mg, 2500 µmol) was added to a solution of N-hydroxysuccinimidyl-6′-(biotinamido)-6-hexanamido hexanoate (NHS-LC-LC-biotin, Pierce) (4.4 mg, 7.750 µmol) in 50 mM sodium phosphate buffer (pH 7.0, 300 µL) and DMSO (300 µL). The reaction was allowed to proceed by stirring overnight at room temperature. The reaction mixture was then purified by HPLC with a gradient of 5–85% acetonitrile in 0.1% TFA/water at a flow rate of 10 mL/min over the course of 25 minutes. Biotin was conjugated to the N-terminal amino groups of the peptides pN7 and N26 with the same procedure. The purified biotin-peptide conjugates were lyophilized, and their identities were confirmed via MALDI-TOF mass spectrometry (positive mode): biotin-pN1 (VWLSYGG), calculated: 1256.10 Da (MNa^+^), found: 1254.87 Da; biotin-pN7 (VMLFYGG), calculated: 1261.19 Da (MNa^+^), found:1260.49 Da; biotin-pN26 (VLALRGG), calculated: 1138.06 Da (MH^+^), found:1137.78 Da.

### Western Blot Analysis

To assay the transfer of wt Nedd8, UB or UB variants to cullin 3 through the NAE-Ubc12 cascade, 5 µM HA-Nedd8, HA-UB or UB variants with an N-terminal HA tag was incubated with 1 µM NAE, 1 µM Ubc12 and 1 µM cullin in the presence of 1 mM ATP, 10 mM MgCl_2_ and 50 µM DTT in the TBS buffer for 1 hour at room temperature. 20 µL of the reaction mixture were loaded on a 4–15% SDS-PAGE gel (Bio-Rad). After electrophoresis, the protein bands were electroblotted onto a piece of polyvinylidene fluoride membrane (Bio-Rad). The membrane was blocked with 3% BSA in TBS buffer for 1 hour followed by incubation with 3% BSA in TBS buffer containing 1∶500 diluted 200 µg/mL anti-HA antibody (Santa Cruz Biotechnology) and 1∶10,000 diluted anti-mouse horseradish peroxidase conjugate (Pierce) for 1 hour, respectively. The membrane was then washed 5 times with TBS-T buffer and 5 times with TBS buffer followed by detection with the ECL luminescent detection kit (GE Healthcare).

To assay the transfer of biotin conjugated peptides to NAE, Ubc12 and cullin, 5 µM biotin labeled peptide, 1 µM NAE, 1 µM Ubc12 and 1 µM cullin were incubated with 1 mM ATP, 10 mM MgCl_2_ and 50 µM DTT in the TBS buffer for 1 hour at room temperature before SDS-PAGE and membrane blotting. The membrane was blocked with 3% BSA in TBS buffer for 1 hour followed by incubation with 3% BSA in TBS buffer containing 1∶10,000 diluted 1 mg/mL streptavidin-horse radish peroxidase conjugate (Pierce) for 1 hour. The membrane was then washed 5 times with TBS-T buffer and 5 times with TBS buffer followed by detection with the ECL luminescent detection kit.

### Peptide Inhibition of Nedd8 Transfer through the NAE-Ubc12 Cascade for Cullin Modification

To assay the inhibition of Nedd8∼NAE thioester formation by the peptides, varying concentrations of the peptides of 10, 20 and 50 µM were incubated with 1 µM NAE in the presence of 1 mM ATP and 10 mM MgCl_2_ in TBS buffer for 1 hour at room temperature. 5 µM HA-Nedd8 was added and the reaction mixtures were incubated for another 15 min to allow Nedd8 transfer to NAE. The reactions were analyzed by SDS-PAGE and Western blotting probed with a mouse anti-HA antibody.

To assay the inhibition of Nedd8 transfer to cullin by the peptides, varying concentrations of the peptides of 5, 10, 20, 50 100 µM and 200 µM were incubated with 0.5 µM NAE, 0.5 µM Ubc12 and 1 µM cullin for 1 hour in the TBS buffer containing 1 mM ATP, 10 mM MgCl_2_ and 50 µM DTT. 5 µM HA-Nedd8 was then added and the Nedd8 transfer reaction was allowed to proceed for 15 min before SDS-PAGE and Western blot analysis. To probe for HA-Nedd8 conjugated proteins, the blot was developed by sequential incubation with 3% BSA in TBS buffer (pH 7.5) containing 1∶500 diluted 200 µg/mL mouse anti-HA antibody (Santa Cruz Biotechnology) and 1∶10,000 diluted anti-mouse antibody - horseradish peroxidase conjugate (Pierce) for 1 hour, respectively.

### Modeling of the NAE Recognition of the C-terminal Sequences of UB Variants from Phage Selection

NAE-peptide models were generated on the basis of the Nedd8-NAE-ATP complex (PDB ID 1R4N) by replacing the native residues 71–75 with the residues of the respective peptides. *In silico* site directed mutagenesis and optimization of the side chain and occasional main chain interactions were carried out with the program COOT [36].
